# Microcirculatory Differences in Children With Congenital Heart Disease According to Cyanosis and Age

**DOI:** 10.3389/fped.2019.00264

**Published:** 2019-07-01

**Authors:** Rafael González, Javier Urbano, María J. Solana, Mónica Hervías, Ana Pita, Rosario Pérez, Reyes Álvarez, Enrique Teigell, Juan-Miguel Gil-Jaurena, José Zamorano, Adolfo Sobrino, Jesús López-Herce

**Affiliations:** ^1^Service of Paediatric Intensive Care, Gregorio Marañón General University Hospital, Madrid, Spain; ^2^Mother and Child Health and Development Network (REDSAMID), Madrid, Spain; ^3^Department of Paediatrics, School of Medicine, Universidad Complutense de Madrid, Madrid, Spain; ^4^Paediatric Anaesthesia, Gregorio Marañón General University Hospital, Madrid, Spain; ^5^Paediatric Cardiac Surgery, Gregorio Marañón General University Hospital, Madrid, Spain; ^6^Paediatric Hemoperfusionist, Gregorio Marañón General University Hospital, Madrid, Spain; ^7^Paediatric Cardiology, Gregorio Marañón General University Hospital, Madrid, Spain

**Keywords:** microcirculation, sidestream dark field imaging, congenital heart disease, children, cyanosis

## Abstract

**Background:** Congenital heart disease (CHD) is one of the main causes of morbidity and mortality in children. Microcirculatory changes in CHD patients have previously been investigated using a variety of techniques. Handheld videomicroscopy enables non-invasive direct visualization of the microcirculatory bed. The aim of our study was to determine if there are microcirculatory differences among CHD patients based on age and the presence of cyanosis.

**Methods:** A prospective observational study was carried out. Patients with CHD undergoing corrective surgery were evaluated after anesthetic induction prior to surgery. Microcirculation was evaluated using sidestream dark field (SDF) imaging. Hemodynamics and respiratory, biochemical, and tissue perfusion parameters were analyzed.

**Results:** A total of 30 patients were included, of whom 14 were classified as cyanotic and 16 as non-cyanotic. Cyanotic patients had a higher total vessel density (TVD) (*p* = 0.016), small vessel density (*p* = 0.004), and perfused small vessel density (*p* = 0.013), while their microvascular flow index (MFI) was lower (*p* = 0.013). After adjustment for age and PaO_2_, cyanotic patients showed increased TVD (*p* = 0.023), and small vessel density (*p* = 0.025) compared to non-cyanotic patients but there were no differences on the MFI. Age was directly correlated with total MFI (spearman's rho = 0.499, *p* = 0.005) and small vessel MFI (spearman's rho = 0.420, *p* = 0.021). After adjustment for the type of CHD (cyanotic vs. non-cyanotic) patients with MFI and small MFI vessels <3 were younger than those with values ≥3 (*p* = 0.033 and *p* = 0.037).

**Conclusions:** SDF-based evaluation of microcirculation in CHD patients showed that patients with cyanotic defects had higher vascular density, as compared to patients with non-cyanotic defects. Younger patients were more likely to have a low MFI regardless of their type of CHD.

## Introduction

The microcirculation involves those small blood vessels in which oxygen is released to the tissues. It includes small arterioles, capillaries, and venules. As cellular functioning relies on an adequate supply of oxygen and nutrients, microcirculatory disfunction might result in organ failure, especially in critically ill patients (1).

**Figure 1:**
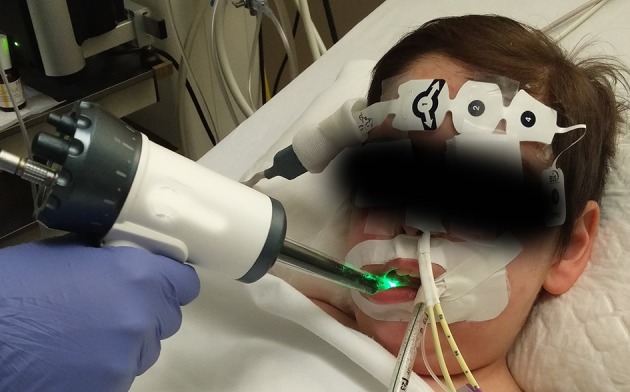
After removal of secretions, sidestream dark field imaging device is steadily applied to the sublingual mucosa to obtain video sequences of the microcirculatory bed.

Different videomicroscopy techniques have been used for the evaluation of microcirculation in adult patients and children, as videomicroscopy enables direct *in vivo* observation of the microvascular vessel network. Sidestream dark field (SDF) imaging consists of a hand-held device that illuminates tissues with a pulsed green light (Figure 1). Light is absorbed by hemoglobin-containing cells, and the light reflected is recorded using a magnification lens system. Video sequences in which microcirculatory blood vessels are outlined by the cells running through them are recorded. Based on these recordings, a variety of microcirculatory parameters can be evaluated (2). Microvascularity of the sublingual mucosa has been proposed as an ideal place to measure microcirculation in critically ill patients. It shares a common embryogenic origin with the digestive mucosa, and it is easily accessible, especially in patients under sedation. Some authors have identified differences between sublingual and other microcirculatory beds in critically-ill patients (3, 4). However, a videomicroscopy-based assessment of microcirculation has been proven as an useful technique in the prediction of outcomes that may guide therapeutic decisions in several diseases in adults (5–9).

In children, most videomicroscopy microcirculation studies have been performed in neonatal population (10). Vascular changes as an adaptation to post-natal life have been a focus of intensive research in studies based on microcirculation imaging techniques (11–15). Some studies have also revealed that microcirculation is impaired during the course of several acute diseases in childhood. As seen in adults, in children microcirculatory acute alterations have been described as predictors of adverse outcomes or responses to treatment in different clinical situations, including severe sepsis, therapeutic hypothermia after cardiac arrest, or respiratory failure (16–20). In addition to acute changes, chronic microcirculatory changes have been described in the childhood of ex-premature children and patients with diabetes mellitus (21, 22). The mechanisms triggering these changes are not clearly known. In the same sense, there is great interest to describe how microcirculation might be modified and thus be used as a direct therapeutic target.

Some studies have been performed in healthy adults to establish normal values for a range of microcirculatory parameters (7, 9, 23–25). Assessing microcirculation in children using videomicroscopy is challenging. Videomicroscopy is only feasible in pediatric patients who are cooperative or sedated (26). There are no studies assessing microcirculation in healthy children after the neonatal period. Some studies suggest that vascular density might decrease with gestational age in neonates and with age in the pediatric population (12, 13, 15, 21). It is important to define what are the normal values for the different microcirculatory parameters in healthy children and in children with different diseases. Once normal values for these parameters are described, it will be possible to use these techniques to identify microcirculatory disturbances and guide therapeutic measures in children.

Congenital heart disease (CHD) is a major cause of disease burden and disability in the world. CHD-associated mortality and morbidity is especially high in the pediatric population (27, 28). Although the clinical symptoms of CHD are generally related to a localized anatomical defect both natural evolution and surgical correction might lead to microcirculatory modifications with different clinical significance. Only a few studies have investigated the effects of CHD on microcirculation in the pediatric population. Children with CHD have revealed altered angiogenesis at different stages of growth (29–34).

The microcirculatory alterations caused by some heart defects have also been identified as an adaptive response to chronic hypoxia (35). Increased blood viscosity (36), reduced red cell deformability (37), augmented shear stress in the blood vessels (38), or modification of endothelial-dependent vasodilatation (39) are some of the mechanisms that have been suggested to be involved in microcirculatory changes in cyanotic patients. Most studies proving microcirculatory changes in CHD patients are based on the microscopic analysis of different tissue samples or on alterations in reactivity tests using different techniques (39–42). Niwa et al. proved that congenital heart disease is related to the presence of aortic wall abnormalities at the medial layer (40). Remodeling of the coronary microcirculation has also been described in patients with cyanotic CHD as a mechanism of preservation of flow reserve (41, 42).

Two studies have been carried out to assess microcirculation in pediatric patients with CHD using videomicroscopy, both in the context of corrective surgery (43, 44). Only in one of these studies, microvascular structure was assessed based on whether CHD was cyanotic or non-cyanotic (44). The objective of our study was to describe the microcirculatory system in different groups of patients with CHD. Identifying microcirculatory differences among groups of patients might be useful in the evaluation of response to therapeutic strategies in acute and long-term conditions.

The purpose of this study was to assess potential microcirculatory differences between CHD patients based on age and the presence of cyanosis. To such a purpose, we used non-invasive videomicroscopy. Our hypothesis was that the microcirculatory system of patients with cyanotic CHD would show differences to the system of patients with other congenital heart diseases, as higher vessel density or increased microcirculatory blood flow. These changes might be interpreted as responses to increased oxygen transportation capability in conditions of hypoxia.

## Methods

A prospective observational study was performed between July 2016 and September 2017. Pediatric patients with CHD undergoing corrective surgery with scheduled admission to the Pediatric Intensive Care Unit were recruited. Patients with oral cavity anomalies or difficult airway management were excluded from the study.

The study protocol was approved by the Institutional Review Board of Gregorio Marañon General University Hospital. Written informed consent was obtained from adult participants and from the parents of participants younger than 18 years of age.

Once in the operating room, anesthesia was induced. First, a peripheral venous access was established, inhalatory anesthesia was induced using sevofluorane, patients were intubated, and mechanical ventilation was initiated. Mechanical ventilation using pressure-controlled ventilation was adjusted to achieve normal ventilation. A central venous catheter and an arterial catheter were placed for hemodynamic monitoring and blood sample extraction. Microcirculation was then evaluated.

### Microcirculatory Evaluation

**Figure 2:**
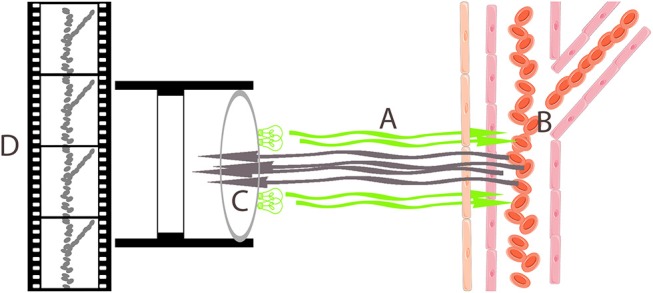
Sidestream darkfield imaging device illuminates sublingual mucosa with a pulsed green light **(A)**. Hemoglobin-containing cells running through blood vessels absorb green light **(B)**, and the reflected light is recorded using a magnification lens system **(C)** that generates a video sequence in which hemoglobin-containing cells are seen as moving gray spots that represent blood vessels **(D)**.

We followed the recommendations proposed by De Backer et al. (45) and Ince et al. (46) in the 1st and 2nd consensus conference on assessing microcirculation. Using Microscan® (Microvision Medical Inc. Amsterdam, The Netherlands), 5 SDF 10-s. video sequences were acquired at both sides of sublingual microcirculation. Before recording was started, oral cavity secretions were removed using a sterile gauze (Figures 1, 2) (2).

Following blinding, deferred recordings were analyzed using AVA Software 3.2® (Microvision Medical Inc., Amsterdam, The Netherlands). After grayscale and contrast were adjusted for to facilitate the identification of vessels, video sequences were stabilized to exclude movement artifacts. Using stabilized video sequences, blood vessels were manually identified. Vessels were manually drawn in each video sequence using a digital tablet Wacom Bamboo Fun Pen & Touch CTH66® (Wacom CO LTD, Saitama, Japan). The course and thickness of each vessel were determined. According to their thickness, vessels were classified into small (those with a caliber below 20 μm) or large (those larger than 20 μm). Table 1 summarizes the microcirculatory variables evaluated. Total vessel density (TVD) was calculated as the image area occupied by vessels. The De Backer score (DB) was also calculated as the number of cross points between vessels and three vertical and horizontal equidistant lines seen in each video sequence. These two parameters (TVD and DB) provide information about vascular density at the microcirculatory level, thereby defining diffusive capacity (46).

**Table 1:** Microcirculatory evaluated parameters.

**Variable**	**Abbreviation**	**Units**	**Utility**
Total vessel density	TVD	mm^2^/mm^2^	Determines the density of vessels in each location. Provides information about diffusive capacity.
De Backer score	DB	n/mm	Determines the density of vessels in each location. Provides information about diffusive capacity.
Microvascular flow index	MFI	Dimensionless (Flow scale 0–4)	Semi-quantitative information about predominant flow velocity.
Perfused vessel density	PVD	mm^2^/mm^2^	Determines the density of vessels with the flow in each location. Gives information about diffusive capacity and capillary distance.
Proportion of perfused vessels	PPV	%	Determines de proportion of vessels with active flow. Provides information about convective flow capacity.
Heterogeneity index	HI		Measures flow variability among the different evaluated locations.

Following vessel identification, flow analysis was performed. First, video images were divided into four quadrants by drawing a horizontal and a vertical midline. Microvascular flow index (MFI) was calculated as the mean value of the predominant type of flow in each quadrant. Flow was semi-quantitatively described as absent (0), intermittent (1), sluggish (2), normal (3), or hyperdynamic (4). After MFI calculation, the flow of each vessel was determined individually according to the same scale. Perfused vessel density (PVD) was calculated as the density of vessels with continuous (sluggish or normal) flow. The proportion of perfused vessels (PPV) was calculated as the percentage of identified vessels in which continuous flow was identified. MFI and PPV provide information about convective blood flow present in the evaluated area and PVD provides information about both diffusive capacity and convective blood flow.

Finally, heterogeneity among images was calculated based on the heterogeneity index (HI), estimated as the highest MFI value minus the lowest MFI value divided by the mean MFI value obtained in each assessment. HI provides information about convective flow variability among the different points evaluated. The higher the HI is, the higher the microcirculatory heterogeneity.

All microcirculatory parameters –except the DB score– were calculated for the totality of vessels and vessels below 20 μm [small vessels (sv)]. Vessels below 20 μm are considered capillaries, especially those below 10 μm in which a single line of red blood cells can be observed.

### Other Measurements

Hemodynamics (arterial blood pressure, central venous pressure and heart rate) and respiratory parameters were also assessed. Tissue perfusion was evaluated by calculating the tissue oxygenation index (TOI) by near infra-red spectrophotometry using INVOS® (Sommanetics, Troy, USA) device. Laboratory values for arterial pH, pO_2_, pCO_2_, SatO_2_, bicarbonate, and lactate were calculated from arterial blood samples.

Clinical data included age, sex, weight, size, body surface area, and diagnosis of congenital heart disease. Congenital heart disease (CHD) was classified into cyanotic and non-cyanotic according to previously published classifications. Table 2 shows the classification of patients as cyanotic or non-cyanotic according to their primary structural defects. Differences in study variables between groups were assessed according to the type of their cardiopathy.

**Table 2:** Classification of patients according to the existence of cyanotic defects.

**Cyanotic congenital heart defects (*n* = 14)**	**Non-cyanotic congenital heart defect (*n* = 16)**
• Double outlet right ventricle + ventricular septal defect (*n* = 3) • Transposition of the great vessels (*n* = 1) • Hypoplastic left heart syndrome (*n* = 2) • Ventricular septal defect + atrial septal defect + overriding aorta (*n* = 2) • Tetralogy of Fallot (*n* = 4) • Transposition of the great vessels + Pulmonary stenosis + ventricular septal defect (*n* = 1) • Total anomalous pulmonary venous return (*n* = 1)	• Atrial septal defects (*n* = 3) • Ventricular septal defect (*n* = 3) • Atrial septal defect + ventricular septal defect (*n* = 1) • Aortic stenosis or insufficiency (*n* = 3) • Aortic coarctation + aortic arch hypoplasia (*n* = 1) • Pulmonary stenosis (*n* = 1) • Coronary fistula (*n* = 1) • Atrioventricular canal (*n* = 3)

### Statistical Analysis

All data were entered into a database (Supplementary Table 1) and analyzed using SPSS Statistics 22.0 for OsX® (IBM Corp, Armonk N.Y., USA). Continuous variables are expressed as medians and interquartile ranks. A comparison of continuous variables was made using Mann-Whitney and Kruskal-Wallis tests. A multivariate linear regression model was developed to study the interaction between variables revealed to have statistical significance though a univariate analysis. To analyze the correlation between variables, Spearman's Rho was calculated. A *p*-value < 0.05 was considered statistically significant.

## Results

A total of 30 patients were recruited during the period of inclusion, 16 of which were classified as non-cyanotic and 14 as cyanotic (Table 2). As many as 150 SDF microcirculatory video sequences were recorded. Six video sequences were unsuitable for analysis due to poor image quality or the presence of artifacts. Finally, 144 sequences were processed and analyzed.

The clinical characteristics, monitoring, and laboratory values for cyanotic and non-cyanotic patients are compared in Table 3. Cyanotic patients had lower SpO_2_, TOI (brain and somatic), arterial pO_2_, and arterial O_2_ saturation. They also required higher peak inspiratory pressure and FiO_2_. When microcirculatory variables were compared between cyanotic and non-cyanotic patients (Table 4), the cyanotic group had higher TVD, TVDsv, and PVDsv, and lower MFI.

**Table 3:** Clinical characteristics, monitoring, and laboratory values of cyanotic and non-cyanotic patients.

	**Non-Cyanotic (*n* = 16)**	**Cyanotic (*n* = 14)**	***p***
Age (years)	1.86 (0.87–5.57)	0.49 (0.35–4.07)	0.051
Weight (kg)	12.5 (8.5–20.5)	7.0 (5.2–13.9)	0.053
Size (cm)	84.8 (71.6–116.8)	65.5 (59–101.3)	0.051
BSA (m2)	0.55 (0.42–0.81)	0.36 (0.3–0.63)	0.056
Heart rate (bpm)	102 (92–108)	108 (88–127)	0.334
SAP (mmHg)	79 (65–87)	74 (59–90)	0.519
DAP (mmHg)	42 (37–50)	38 (33–47)	0.212
MAP (mmHg)	58 (49–65)	51 (40–61)	0.349
CVP (mmHg)	8 (7–10)	7 (6–10)	0.711
Temperature (°C)	35.9 (35.7–36.4)	36.1 (35.6–36.6)	0.609
SpO_2_ %	98 (98–99)	93 (86–98)	**0.009**
Brain TOI	76 (68–81)	67 (59–73)	**0.008**
Somatic TOI	89 (80–95)	65 (63–80)	**0.001**
PEEP (cm H2O)	5 (4–5)	5 (4–5)	0.424
PIP (cm H2O)	16 (14–18)	18 (17–20)	**0.026**
FiO_2_ (%)	40 (35–40)	45 (38–59)	**0.026**
Arterial pH	7.39 (7.37–7.47)	7.39 (7.35–7.47)	0.368
Arterial pO_2_	163 (82–218)	67 (51–119)	**0.009**
Arterial pCO_2_	38 (33–41)	38 (35–45)	0.456
Serum bicarbonate	24 (22–24.8)	23.8 (21–27.7)	0.777
Arterial SatO_2_ (%)	97 (96–99)	93 (88–97)	**0.015**
Glucose (mg/dl)	93 (82–100)	90 (80–101)	0.965
Calcium (mmol/l)	1.2 (1.2–1.3)	1.2 (1.2–1.3)	0.661
Lactate (mmol/l)	0.9 (0.7–1.0)	1 (0.8–1.1)	0.354
Hemoglobin (gr/dl)	11.1 (10.2–11.4)	11.7 (10.4–13.1)	0.182
Hematocrit (%)	33 (32–35)	34 (29–36.2)	0.756

*BSA, Body surface area; SAP, Systolic arterial pressure; DAP, Diastolic arterial pressure; MAP, Mean arterial pressure; CVP, central venous pressure; SpO_2_, Peripheral oxygen saturation; TOI, Tissue oxygenation index; PEEP, Positive end expiratory pressure; PIP, Peak Inspiratory pressure; FiO_2_, inspired oxygen concentration; pO_2_, O_2_ partial pressure; pCO_2_, CO_2_ partial pressure; SatO2, O_2_ saturation. Bold values indicate statistically significant values*.

**Table 4:** Comparison of microcirculatory values between cyanotic and non-cyanotic patients.

	**Non-cyanotic (*n* = 16)**	**Cyanotic (*n* = 14)**	***p***
TVD (mm/mm2)	24.6 (23.2–27.4)	27.2 (25.2–31.2)	**0.016**
TVDsv (mm/mm2)	22.0 (21.0–25.5)	24.8 (23.6–30.4)	**0.004**
DB	15.1 (14.6–16.9)	16.2 (14.7–19)	0.220
PVD (mm/mm2)	23.9 (22.8–26.8)	25.5 (23.7–28.8)	0.135
PVDsv (mm/mm2)	21.0 (20.4–25.2)	24.1 (22.1–28.1)	**0.013**
PPV (%)	98.0 (95–99.9)	96.6 (92.5–98.3)	0.287
PPVsv (%)	98.0 (94.3–99.9)	96.5 (92.1–98.3)	0.337
MFI	3.0 (3.0–3.0)	3.0 (2.9–3.0)	**0.013**
MFIsv	3.0 (3.0–3.0)	3.0 (2.7–3.0)	0.121
HI	0.06 (0.00–0.18)	0.17 (0.03–0.33)	0.119
HIsv	0.00 (0.00–0.34)	0.26 (0–0.54)	0.283

*TVD, Total vessel density; sv, small vessels; DB, De backer score; PVD, Perfused vessel density; PPV, proportion of perfused vessels; MFI, microvascular flow index; HI, heterogeneity index. Bold values indicate statistically significant values*.

A multivariate regression model was developed to examine whether differences in vascular density between cyanotic and non-cyanotic patients were influenced by other factors such as age or ventilation parameters. After adjustment for age and PaO_2_, cyanotic patients showed a mean TVD 3.07 mm/mm^2^ (CI 95% 0.45–5.70) and TVDsv 3.28 mm/mm^2^ (CI 95% 0.44–6.13) higher than non-cyanotic patients (*p* = 0.023 and *p* = 0.025, respectively).

No patient showed MFI nor MFIsv above three. As described in Table 4, in the unifactorial analysis the group of cyanotic patients showed lower MFI than non-cyanotic patients, in the same way, cyanotic patients were more likely to have an MFI <3 as compared to non-cyanotic patients (*p* = 0.012). However, after adjustment for age, no statistically significant differences were observed in MFI between cyanotic and non-cyanotic patients (*p* = 0.765).

**Figure 3:**
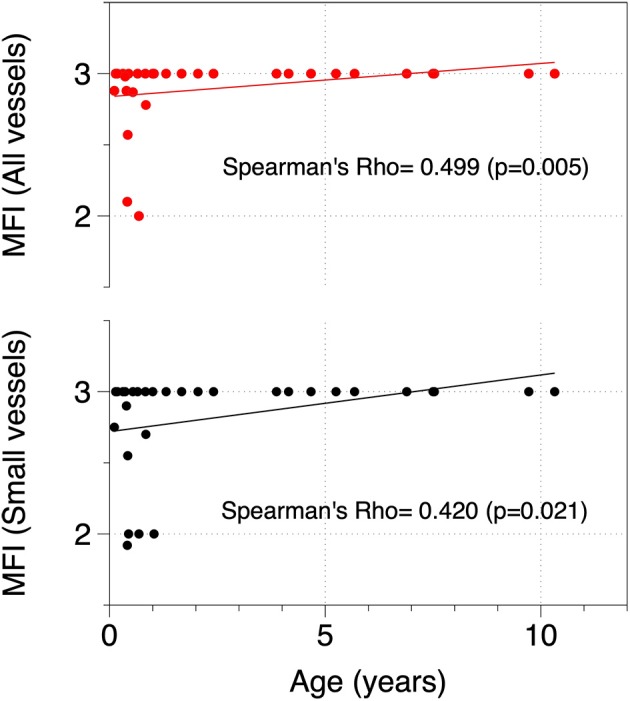
Correlation between age and MFI for all vessels and small vessels. MFI, Microvascular flow index.

The correlation between age and microcirculatory values was analyzed (Table 5). MFI and MFIsv showed a positive correlation with age (Figure 3). No other microcirculatory variables were found to be correlated with age. After adjustment for type of cardiopathy, patients with MFI <3 were 3 years younger (CI 95% 0.26–5.8) than those with MFI ≥3 (*p* = 0.033). The same phenomenon occurred with the MFI of small vessels (those <20 μm). Patients with MFIvp <3 were 2.8 years younger (CI 95% 0.18–5.3, *p* = 0.037) than those with MFIvp of 3.

**Table 5:** Correlation between age and microcirculatory variables.

**Microcirculatory variable**	**Spearman's Rho**	***p***
TVD (mm^2^/mm^2^)	−0.021	0.914
TVDsv (mm^2^/mm^2^)	−0.152	0.421
DB (n/mm)	−0.069	0.717
PVD (mm^2^/mm^2^)	−0.034	0.86
PVDsv (mm^2^/mm^2^)	−0.043	0.82
PPV (%)	0.188	0.319
PPVsv (%)	0.219	0.245
MFI	0.499	**0.005**
MFIsv	0.420	**0.021**
HI	−0.26	0.166
HIsv	−0.185	0.329

*TVD, Total vessel density; sv, small vessels; DB, De backer score; PVD, Perfused vessel density; PPV, proportion of perfused vessels; MFI, microvascular flow index; HI, heterogeneity index. Bold values indicate statistically significant values*.

## Discussion

Increased vessel density has been previously linked to the presence of congenital defects causing systemic hypoxemia. This phenomenon has been described in different organs in studies based on histological examination (30, 31, 34, 47). In our study, we detected microcirculatory differences between cyanotic and non-cyanotic CHD patients using SDF. Thus, cyanotic patients exhibited around 10% more small blood vessels in sublingual microcirculation (venules and capillaries) than non-cyanotic patients (Table 4). Although there were some differences between cyanotic and non-cyanotic patients (especially in age and respiratory parameters), after adjusting for age and p_a_O_2_, multivariate analysis showed that the group of patients with cyanotic CHD had a higher vascular density.

Oxygen transport from blood cells to tissue cells depends mainly on two factors that define microvascular function, namely convective blood flow and diffusive capacity. Diffusive capacity is mainly determined by the density of microcirculatory blood vessels (48). Thus, increased vessel density in patients with low blood oxygen concentrations could be an adaptive mechanism for reducing diffusion distance and improving oxygen transport. This phenomenon has been previously reported by several authors. Gassmann et al. described that Andean patients born after a high-altitude pregnancy had increased vessel density, as compared to those born after a sea-level pregnancy (49). And Gilbert-Kawai et al. showed that Sherpas highlanders in the Everest have greater capillary density than lowlanders (50). All these findings remark that microcirculation adapts to compensate hypoxia.

Several authors have described that vessel density decreases with age in neonates and children (13, 15, 21, 51). In our patients, we have not found a correlation between age and any microcirculatory variable related to vessel density. Conversely, we found that age was correlated with MFI, and younger patients showed a decreased MFI more frequently than older patients did. There might be several explanations for this striking finding. First, we did not find that microvascular vessel density changed in children over time, as described by other authors. However, our population was very different to the populations evaluated in previous studies, as we only included patients with CHD. Second, van Elteren et al. described that preterm neonates show low MFI values at birth, which become normal over time (13). In our study, most patients were not neonates; therefore, it is unlikely that changes in MFI were caused by adaptation to post-natal life. We can speculate that this finding might be related to age-related differences in the effects of anesthetic induction, especially in patients with congenital heart disease. Anesthesia-related changes in both, macro- and microcirculation have been previously described with contradictory results (52–56). Riedijk et al. showed that propofol infusion in children during procedural sedation was related to a decrease in mean blood pressure and an increase in microvascular density. MFI was not altered in their group of patients, but their sample only included patients between 8 and 18 years of age, and the effects of anesthesia in younger patients were not considered (57).

Patients with CHD who need surgery during their first months of life are usually affected by severe anatomic defects in which cardiac output and thus tissue perfusion might be compromised. Conversely, patients in which surgery is performed in later moments are considered to be stable and in them, systemic perfusion is usually conserved. These facts can also explain why in our patients MFI was lower in the younger patients. It is necessary to perform larger studies in which changes on MFI with age could be compared between groups of patients with different severity.

Normal values for the MFI have been previously described for adults, but not for children. Vellinga et al. have used an MFI below 2.6 to differentiate between patients with altered microcirculation and patients with conserved microcirculation (7). Considering that normal values are not defined for children, and that in our study no patient showed any sequence with hyperdynamic flow, we have established a comparison between those showing MFI and MFIsv scores below three and those in which the score was three. In the second group, all measurements performed scored for a normal flow in every quadrant. In our group of patients those with MFI scores below three were younger than those with a score of three. It is necessary to establish what are the normal values for the different microcirculatory parameters in healthy children.

## Limitations

Our study has several limitations. We evaluated microcirculation in a small group of patients representing a wide spectrum of heart defects with a variety of clinical features. The development of the microcirculatory bed is influenced by macroscopic anatomical changes; however, it could also be conditioned by the clinical status of the patient. In this sense, patients with similar heart defects may have very different clinical situations, and the structure of their microcirculatory bed might be very different too. We have not categorized patients according to the severity of preexisting cyanosis. This could have been useful to determine if the intensity of microvascular remodeling is related to the degree of cyanosis. As patients were evaluated at the time of surgery once they were anesthetized, intubated, and mechanically ventilated, we could not determine the baseline degree of cyanosis of each patient.

Additionally, we evaluated microcirculation only in patients with congenital heart disease at the time of surgery. Microcirculation might differ under the effects of anesthesia and mechanical ventilation, to the observed without anesthesia. Larger studies including healthy children and children with other congenital heart diseases are needed to characterize the effects of heart disease on microcirculation in childhood.

Image acquisition and analysis were performed according to published recommendations (45, 46). During image acquisition, researchers tried to avoid common artifacts as those corresponding to secretions, high pressure or those related to inadequate illumination. In the analysis phase, videos not fulfilling quality criteria were excluded from evaluation.

Microcirculatory evaluations—which involved video sequence acquisition and deferred evaluation—were performed by a single investigator (R.G.). This could also point to a possible source of bias. Formal certified training for the different videomicroscopy microcirculatory evaluation techniques has not been established, although some initiatives have been undertaken in this sense (58). Ideally, analysis of acquired video sequences should be performed automatically. Yet, the reliability of fully automated SDF imaging has not been verified, and most guidelines recommend the use of off-line software-aided analysis. R.G. was responsible for image acquisition and off-line analysis of more than 500 SDF video sequences collected from previously published studies (26, 59). The large experience of the researcher in image acquisition and analysis might minimize bias. Video sequences were also anonymized, and time-point blinding prevented analysis-related bias.

## Conclusions

Children with cyanotic congenital heart defects exhibit increased vessel density as compared to those with non-cyanotic congenital heart defects. Among patients with congenital heart defects, it is more frequent to have decreased MFI in younger patients.

## Ethics Statement

The study protocol was approved by the Institutional Review Board of Gregorio Marañon General University Hospital. Written informed consent was obtained from adult participants and from parents of participants younger than 18 years of age.

## Author Contributions

SDF image acquisition and analysis was performed by RG, JU, MS, and JL-H. Monitoring variables were registered by MH and ET. Blood samples were obtained and analyzed by RP and JZ. AP, RA, AS, and J-MG-J were responsible for gathering patient information and diagnoses. RG was responsible for manuscript drafting. All authors participated in the study design and data interpretation, and involved in the evaluation of the manuscript and design of the final version.

### Conflict of Interest Statement

The authors declare that the research was conducted in the absence of any commercial or financial relationships that could be construed as a potential conflict of interest.
